# P153: The prevalence of infection among food vendors in Birim Central of Ghana, West Africa, using biological indicators

**DOI:** 10.1186/2047-2994-2-S1-P153

**Published:** 2013-06-20

**Authors:** S Boison

**Affiliations:** 1 Environmental Health, Syband Ngo, Accra, Ghana

## Introduction

The operation and patronage of fast-food joints, restaurants, and chop bars have increased and become common in the Ghanaian community, especially, in urban areas. Despite the benefits derived from these food joints, their operation raises public health issues, since food vendors could be a major transmission source for infections. Poor knowledge of hygiene and practices in food service establishments can contribute to outbreaks of foodborne illnesses.

## Objectives

This study sought to determine the prevalence of infection among food vendors in Birim Central of Ghana, West Africa, using biological indicators. The main purpose of the study was to estimate the level of infection among food vendors in the Birim Central of Ghana and to initiate proper measures to control and prevent the spread of the infection.

## Methods

A cross-sectional study was conducted in Birim Central. Data were collected using the results of the laboratory tests done on 4243 food vendors from 16^th^ January to 15^th^ February 2013. The sampled vendors were made up of 94.53% females and 5.47% males.

Convenient sampling was used to select 4243 food vendors from the Birim Central which had a population of about 7000 food vendors. The biological profiles of urine and blood samples were developed from the vendors sampled. The blood was tested for widal test (O antegen) levels whilst the urine was tested for blood and protein levels. The cut-off points for determining agglutination was titres above 1/160. Those whose samples agglutinated above 1/160 dilution of the widal test were considered to have high antibody. Proportions were calculated for the prevalence of infections among food vendors.

## Results

The results showed that 2.85% of the sampled vendors had hand infection. It showed that 31.88% did not know the proper method of washing their hands. The study again showed that 4.22% had protein in their urine and 7.78% had blood in the urine. Again, 16.64% who had above 1/160 in the widal test (O antegen) had high anti-body.

## Conclusion

There is prevalence of high anti bodies signified by high widal test. Most of the vendors demonstrated unacceptable hand washing technique which could be the cause of hand infections among the vendors.

## Disclosure of interest

None declared

